# Recent Progress on Chemical Constituents and Pharmacological Effects of the Genus *Nigella*

**DOI:** 10.1155/2020/6756835

**Published:** 2020-06-19

**Authors:** Yun Niu, Li Zhou, Lijun Meng, Sitan Chen, Changyang Ma, Zhenhua Liu, Wenyi Kang

**Affiliations:** ^1^National R & D Center for Edible Fungus Processing Technology, Henan University, Kaifeng 475004, Henan, China; ^2^Joint International Research Laboratory of Food & Medicine Resource Function, Kaifeng 475004, Henan, China; ^3^Kaifeng Key Laboratory of Functional Components in Health Food, Kaifeng 475004, Henan, China

## Abstract

Seeds of the genus *Nigella* plants as folk medicine are often used to prevent and treat asthma, diarrhea, dyslipidemia, and other diseases around the world. Pharmacological researches showed that seed extract and seed oil have antibacterial, antioxidant, hypoglycemic, and hepatoprotective effects which attributed to their bioactive constituents such as alkaloids, saponins, flavones, and phenols. This paper has covered recent progresses on chemical and pharmacological researches on these plants, including their compounds and pharmacological effects. It was found that the chemical component researches were focused on the seed oil. Therefore, more attention should be paid to the profile of the whole constituents in the seeds.

## 1. Introduction

Plants of the genus *Nigella* originate from family Ranunculaceae, which contain about twenty species, mainly distributed in the Mediterranean area. Two species are cultivated in China, *Nigella damascena* L. and *Nigella glandulifera* Freyn et Sint. *N. damascena* originates in southern Europe and is cultivated as ornamental plants in China. *N. glandulifera* is mainly found in Xinjiang and Tibet [1]. In addition, *N. sativa*, known as “black cumin,” is distributed in Southern Europe, southwest Asia, North Africa, and mainly in Pakistan and Egypt [2].

Owing to the large quantities of nutritional and medicinal constituents, seeds of genus *Nigella* plants are widely used in food preparations and medicine [2]. *N. glandulifera* seeds are a common Uyghur medicine, which have galactagogue, diuretics, bronchodilator, and analgesic activities and cure bronchial asthma, edema, and urinary calculus [3]. *N. sativa* seeds have a long history in usage as the wind dispelling agent, diuretics, and insect repellent [4]. *N. damascena* seeds are widely used as aromatic agent in bread and cheese, and it is also used in folk medicine to treat menstrual disorders [5]. In view of sufficient literatures on *Nigella* seeds, here we have summarized the active constituents and their pharmacological activities to provide theoretical elucidation for further utilization.

## 2. Chemical Constituents

Due to the pharmacological activities of *Nigella* seeds, phytochemical studies were conducted to find all kinds of natural compounds. Over the past few decades, the chemical composition investigations were mainly focused on oils. However, literatures have revealed that the seeds of *Nigella* genus contained many secondary metabolites such as steroids, flavones, saponins, alkaloids, and phenols. All compounds from the genus *Nigella* and their references are listed in Table 1.

### 2.1. Alkaloids

**Figure 1:**
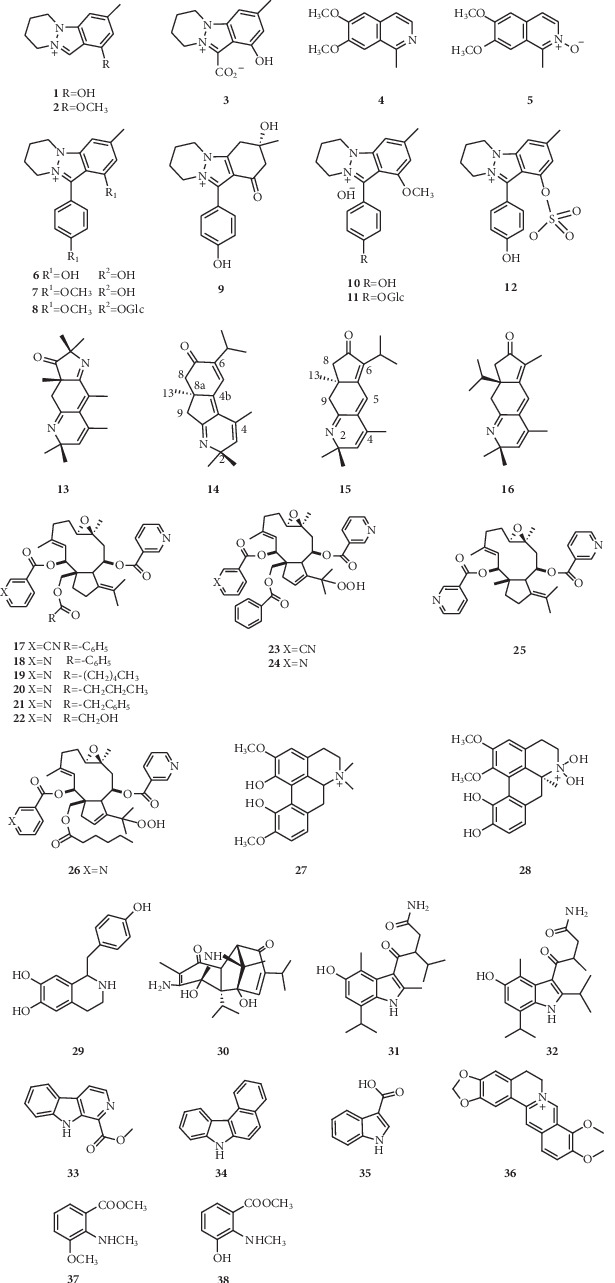
Akaloids isolated from *Nigella* genus.

Thirty-eight alkaloids (**1–38**) (Figure 1) were derived from seeds of *Nigella* genus plants. Natural alkaloids including the indazole ring are infrequent and hitherto identified only ten analogs. Compound **3**, first natural alkaloid with an indazole ring, was isolated and identified by Rahman in 1985 [3]. Compound **8** was the first natural alkaloidal glycoside. Containing a highly cross-ring conjugated system, compound **13** was classified as pyrroloquinoline alkaloid, and compounds **14**–**16** were classified as norditerpenoid alkaloids according to their unique skeletons. The configuration at C-8a for compounds **14** and **15** was *S*, which was established by comparison of their experimental electronic circular dichroism (ECD) spectra with quantum chemical ECD calculation. Compounds **14–16** was separated and purified by pH-zone-refining countercurrent chromatography, and compound **13** was isolated by high-speed countercurrent chromatography [11]. Novel dolabellane-type diterpene alkaloids **17–26** were also rare in nature [12–14]. In addition, compounds **30–32** with unprecedented skeletons could be speculated to derive from the dimerization of thymoquinone (TQ) [18]. Notably, to date, natural indazole-type alkaloids have only been obtained from species of the genus *Nigella* and therefore considered as possible taxonomic markers in *Nigella* genus plants.

### 2.2. Flavonols

**Figure 2:**
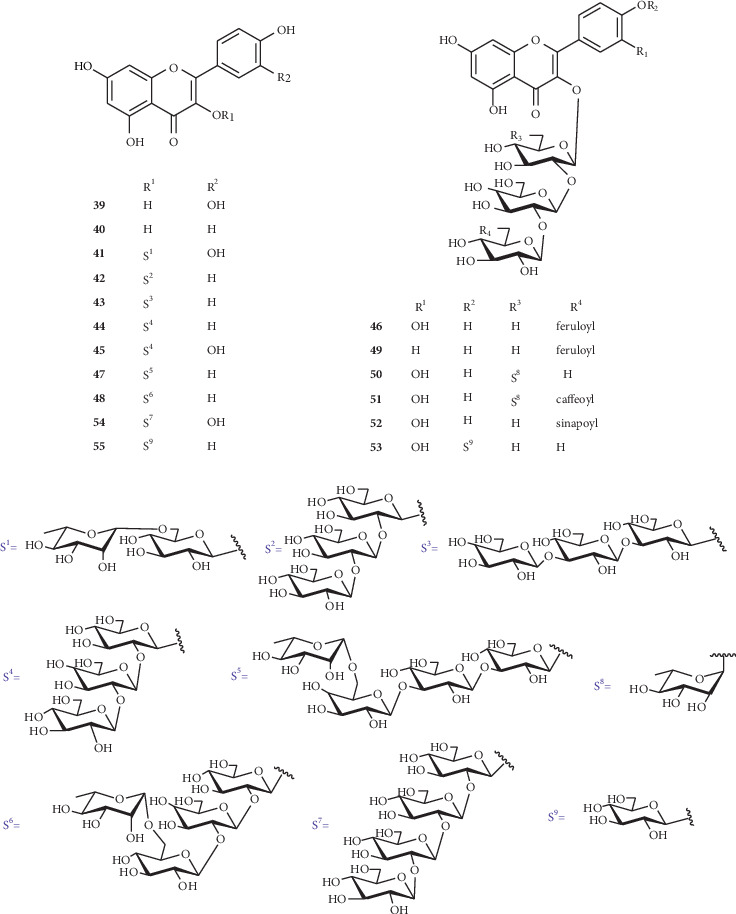
Flavonols and sugar residues isolated from *Nigella* genus.

Seventeen flavonols (**39–55**) (Figure 2) have been obtained from the seeds of *Nigella* genus plants, which are mainly derived from *N. glandulifera* and *N. sativa*. The localizations and classes of the glycosyl or acetyl groups are shown as follows.

### 2.3. Triterpenoids

**Figure 3:**
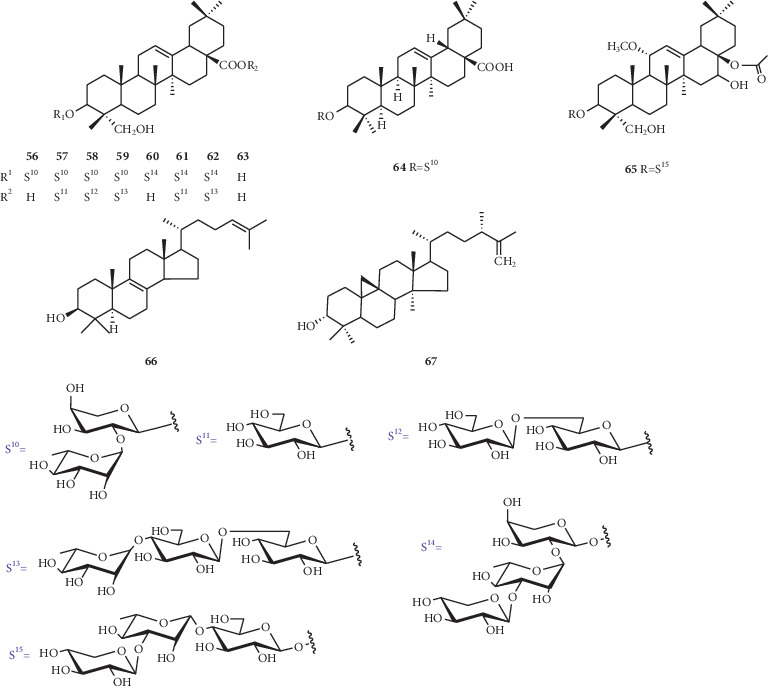
*Triterpenoids* and sugar residues isolated from *Nigella* genus.

The seeds of this genus are rich in triterpenoid components, mostly with hederagenin as the basic skeleton. There are ten compounds (**56–65**) with hederagenin as their mother nucleus from genus *Nigella*. The linkage pattern and locations of glycosyl moieties are shown in Figure 3.

### 2.4. Steroids

**Figure 4:**
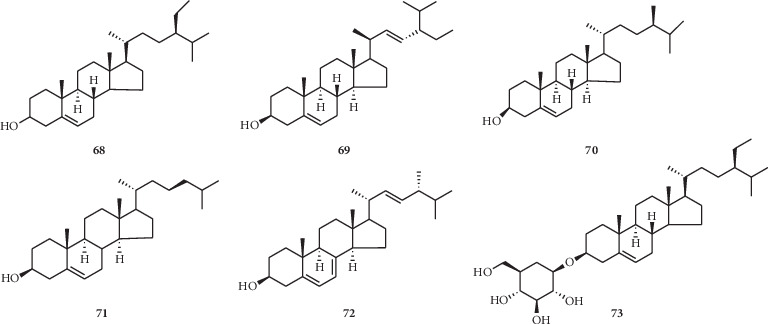
Steroids isolated from the *Nigella* genus.

There are six steroids (**68–73**) (Figure 4) isolated from the seeds of this genus, mostly existing in *N. sativa* seeds oil.

### 2.5. Phenols

**Figure 5:**
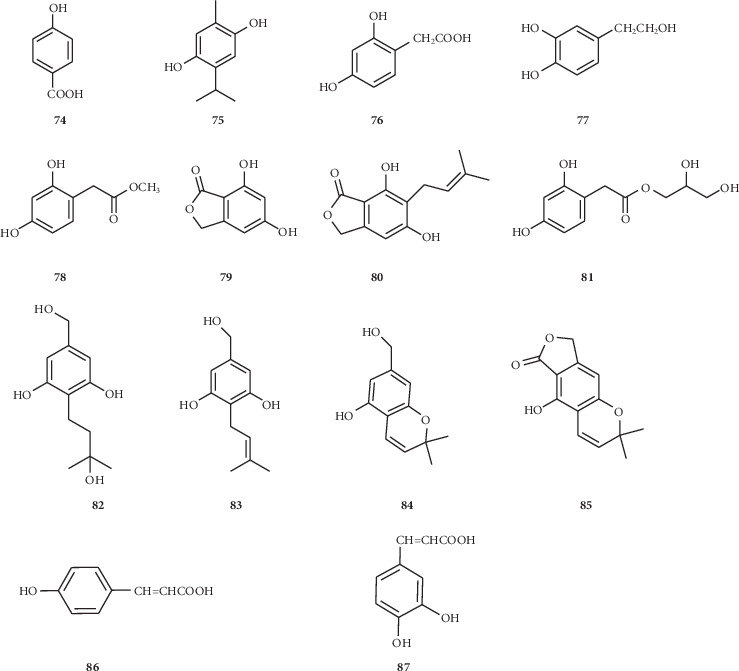
Phenols isolated from the *Nigella* genus.

Twelve phenolic compounds (**74–87**) (Figure 5) were isolated from the seeds of this genus. Compounds **80** and **85** were isobenzofuranone derivatives. Compounds **82–84** contain hydroxymethyl moiety. Compounds **84** and **85** both observed an oxygen bridge between C-8a and C-2, an (Z)-3-methylbutenyl unit of the tetrasubstituted benzene ring.

### 2.6. Others

**Figure 6:**
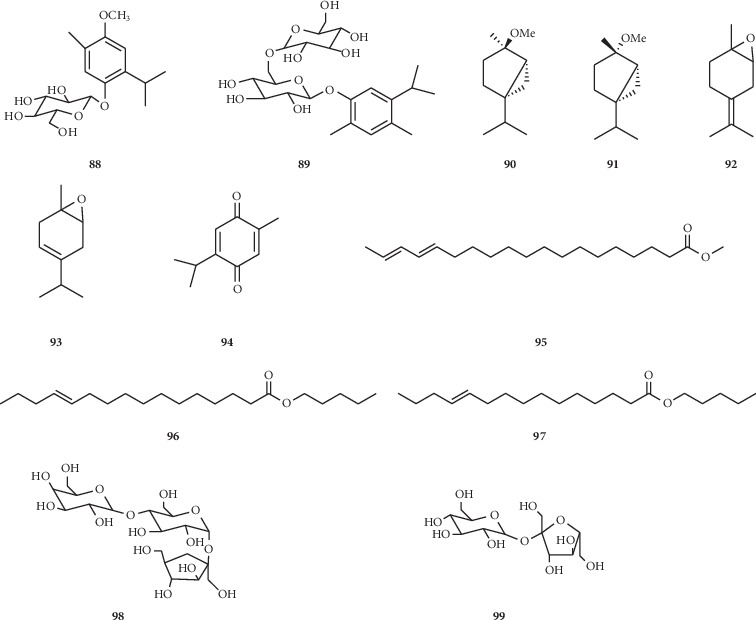
Monoterpenoids, saccharides, and lipids isolated from the *Nigella* genus.

In addition, other categories compounds were obtained from *N. glandulifera,* such as monoterpenoids **88–89** [24, 37] and saccharides **98** and **99** [33]. Compounds **95–97** [40] were identified as novel lipids with long-chain aliphatic nature and unsaturated double bond from *N. sativa*. And, monoterpenoids **90–94** were derived from the volatile oil of *N. sativa* [38] (Figure 6).

The seeds of *Nigella* plants are affluent in lipids and oil, with the content of lipids at 35%–42% and the volatile oil accounting for 1.5%. As the main active constituent in volatile oil, compound **99** (TQ) was clarified possessing many pharmacological activities [39]. Hao and Ren [41] had identified 10 fatty acids from the seeds of *N. glandulifera*, among which linoleic acid was the most abundant. In addition, oleic acid, palmitic acid, and three glyceryl hexadecanoate were also found from the seeds of *N. glandulifera*.

Moreover, two polysaccharides were determined in *N. sativa* seeds. BCWSP with average molecular weight about 800 kDa mainly contained arabinose (5.83%), galactose (5.76%), xylose (3.19%), glucose (3.18%), rhamnose (2.74%), and mannose (2.28%). And, the yield of the polysaccharides was 5.18% [42]. With the molecular weight determined as about 95 kDa, BCPP exhibited the content of rhamnose : arabinose : xylose : galactose : glucose in a ratio of 29 : 42 : 2 : 24 : 3 as main sugar residues and yielded approximately 1.5% [43].

## 3. Pharmacological Effects

Many researches have been carried out, especially during the past two decades, on the effect of *Nigella* seeds extracts, oils, and isolated compounds in vivo or in vitro owing to their wide range of biological activities. As a most abundant oil constituent, thymoquinone has antioxidant, anti-inflammatory, immunomodulatory, antihistaminic, antimicrobial, and antitumor effects. In addition, many researches have indicated that thymoquinone had various pharmacological activities, proving to be responsible for oil activities. In addition, some clinical trials have been conducted on eligible patients to evaluate the effects of *N. sativa* seeds on glycemic control, inflammation, oxidative stress, and so on [44]. And, we have summarized the characteristics of several human studies regarding the effect of *Nigella sativa* seeds in Table 2.

### 3.1. Antibacterial Activity

Many studies have discussed the antibacterial efficacy of *N. sativa* seeds extracts, as well as oil and compounds. Ayeza et al. [52] found that the activity of the ethanolic extract was better than that of the methanolic extract against *Escherichia coli*, *Vibrio parahaemolyticus*, and *Bacillus cereus* (MIC value 0.25 mg/ml), while the methanolic extract was more active against *Listeria monocytogenes*. Another study indicated that the methanolic extract of *N. sativa* seeds had a stronger bactericidal activity than antibiotic ciprofloxacin against *Corynebacterium*, *Staphylococcus aureus*, and *S. viridians* by the disc diffusion assay [53]. As the antibiotic resistance is becoming a crucial problem, *N. sativa* seeds also have been tested against the resistance strains. The methanol extract of *N. sativa* seeds showed the MIC value in the range of 0.39–1.5 mg/mL (*p* < 0.001) against methicillin-resistant *S. aureus* (MRSA) strains. Subsequently, in this research, when combined with *N. sativa* methanolic extract, cefoxitin inhibited MRSA strains at 0.312 *μ*g/mL, which was 64 times lower than that of ceftoxitin alone. Development of this synergism might be a significant way to enhance the activity of ceftoxitin to alleviate the resistance process [54].

In addition, *N. sativa* essential oil exhibited an activity on *S. aureus*, *E. coli*, and *Pseudomonas aeruginosa* with MIC values of 2.5, 8, and 2 *μ*g/mL respectively, while the streptomycin group showed an MIC value in range of 20–100 *μ*g/mL [55]. It was found that thymoquinone (TQ), which is the major constituent of the oil, exhibited positive effect against *Clostridium difficile* with the MIC at 10–40 *μ*g/mL, while TQ showed activities against other microorganisms (*C. perfringens*, *Bacteroides fragilis*, and *B. thetaiotaomicron*) with the MIC value at 80–160 *μ*g/mL [56]. It was also found that compounds **93** and **94** showed an activity against *S. aureus* with the zone of inhibition in the range of 9–11 mm by the agar diffusion technique with amphotericin B as the positive control [40].

### 3.2. Antifungal Activity

In addition, *N. sativa* essential oil was also a good fungal inhibitor, exhibiting activities on *Microsporum gypseum* (diameter of inhibition zone: 38 mm), *Trichophyton rubrum* (20 mm), and *T. simii* (35 mm), compared with ketoconazole (10, 15, and 32 mm, respectively) [57]. Another finding was that *N. sativa* essential oil could inhibit *Candida albicans* (MIC value at 2 *μ*g/mL) and *Aspergillus fumigatus* (5 *μ*g/mL) in comparison with fluconazole (5 *μ*g/mL) [55]. It was also found that thymoquinone exhibited an activity against *Trichophyton mentagrophytes*, *Microsporum canis*, and *M. gypseum* by the disk diffusion method (with inhibition zone diameter >50 mm), while ketoconazole was in the range of 28–31 mm [58]. Owing to their antifungal effect, *Nigella sativa* oils can be considered for further investigations to develop antifungal agents.

### 3.3. Antiparasitic Activity

*N. sativa* aqueous extract had an activity against human *Blastocystis hominnis*, with an equivalent effect of metronidazole at 500 *μ*g/mL [59]. The methanolic extract of *N. sativa* seeds significantly suppressed the *Plasmodium yoelli nigeriensis* growth by 94% at a dosage of 1.25 g/kg body weight (*p* < 0.05) in the mice, while chloroquine showed 86% inhibitory rate in comparison with the untreated group. Moreover, *P. yoelli* infection led to a significant (*p* < 0.05) decline in the activities of catalase (CAT), glutathione-S-transferase (GST) and superoxide dismutase (SOD). The extract of *N. sativa* seeds could restore the activities of these parameters to near normal [60]. In a recent study, it was found that thymoquinone could inhibit the growth of piroplasm parasites in vitro with IC_50_ values of 35.41, 7.35, 0.28, 74.05, and 67.34 *μ*M for *Babesia bovis*, *B. bigemina*, *B. divergens*, *Theileria equi*, and *B. caballi*, respectively [61].

### 3.4. Antioxidant Activity

Some recent studies found that volatile oil, polysaccharide, and extracts in *Nigella* plant seeds possessed an antioxidant activity. Antioxidant enzymes including CAT, SOD, GST, glutathione (GSH), and glutathione peroxidase (GSHPx) were the major constituents of the antioxidant system in most cells. And, antioxidant enzymes play a positive role in neutralizing the free radical-induced oxidative injury. It was found that pretreatment with thymoquinone could attenuate the inhibitory effect of H_2_O_2_ on the GSH level and superoxide dismutase activity. Moreover, the activation of the nuclear factor erythroid-2-related factor 2/heme oxygenase-1 (Nrf2/HO-1) pathway in H_2_O_2_-induced human retinal pigment epithelium (ARPE) cells was enhanced by thymoquinone. The results indicated that thymoquinone could protect ARPE cells from H_2_O_2_-induced oxidative stress through the Nrf2/HO-1 pathway [62]. Furthermore, a recent study was conducted on forty patients with Hashimoto's thyroiditis to evaluate the effect of powdered *N. sativa* seeds (1 g/day for 8 week) on oxidative stress. The result showed that treatment with *N. sativa* could increase the serum total antioxidant capacity (TAC) and SOD and reduce the MDA concentrations [45].

Three terpenes **95–97** isolated from *N. sativa* seeds showed effect to inhibit the oxidative stress in human skin WS-1 fibroblasts, with IC_50_ of 0.32, 0.005, and 0.43 mM, respectively [38]. A study was conducted on the polysaccharide fraction (named BCWSP) to test the antioxidant effect, and the results showed that the highest DPPH radical-scavenging activity (63.25%) was recorded at 1 mg/mL and the lowest (21.59%) was obtained at 0.2 mg/mL treated with polysaccharide BCWSP. The study revealed polysaccharide BCWSP possessed an antioxidant activity in a concentration-dependent manner [42].

### 3.5. Antidiabetes

Plant-derived medications have become a significant manner to treat the diabetes to reduce the adverse effect. Numerous studies have been conducted on the hypoglycemic activity of alkaloids of *N. glandulifera* and oils of *N. sativa*. It was found that *N. sativa* seeds oil could downregulate the expression of the insulin receptor gene and upregulate the expression of insulin-like growth factor-1 (IGF-1) and PI3K compared with the control group. In addition, parameters like blood glucose, liposome composition, and tumor necrosis factor-*α* (TNF-*α*) were also reduced by *N. sativa* seed oil. [63]. It was also found that *N. sativa* seeds oil could inhibit *α*-glucosidase with IC_50_ 0.55 mg/mL compared with acarbose (0.53 mg/mL) and thymoquinone (0.65 mg/mL) [64]. Another study was made to evaluate the activity of *N. sativa* seeds oil on type 2 diabetes mellitus (T2DM) patients. Results indicated that *N. sativa* oil was equivalent to metformin in losing weight. *N. sativa* seeds oil also demonstrated a reduction comparable with metformin on some lipid profile parameters like high-density lipoprotein (HDL), low-density lipoprotein (LDL), total cholesterol (TC), and insulin resistance (IR) [46].

Alkaloids **13–16** isolated from *N. glandulifera* showed protein tyrosine phosphatase 1B (PTP1B) inhibitory activity with no obvious toxicity to A431 cells at 100 *μ*M [11]. Compounds **14–16** could activate the phosphatidylinositide 3-kinase-protein kinase B (PI3K/Akt) phosphorylation and downregulate the expression of PTP1B protein in L6 moytubes. Western blot results also demonstrated that compounds **14–16** could inhibit PTP1B by activating the insulin receptor substrate-1/Akt and promote the glycogen synthesis via Akt-mediated glycogen synthase kinase 3 phosphorylation [65]. These alkaloids could serve as leading compounds for the investigation of antidiabetic medicine.

### 3.6. Anticancer Activity

The intrinsic pathway of apoptosis is under the regulation of proapoptotic (such as Bax) and antiapoptotic (such as Bcl-2) genes. Caspases and survivin are also responsible for the execution of apoptosis. Recently, a report was conducted to test the antiproliferative and apoptotic activities of potent *N. sativa* (named P1). Results revealed a concentration-dependent inhibition in MCF-7 cells. There was a reduction in the cell count with the raise in the concentration demonstrated by phase-contrast microscopy images. P1 could upregulate BAX and CASPASE-3 together with downregulation in survivin and BCL-2 gene expression [66]. Several preclinical researches indicated that TQ could induce apoptosis and restrain hepatocellular carcinoma (HCC) progression via different pathways. These results suggested potential applications of TQ for HCC treatment in clinical practices [67–71]. TQ was a perspective chemotherapeutic drug on gliomas and glioblastomas as it could cross the blood-brain barrier with selective virulence for glioblastoma cells in comparison with primary astrocytes [72]. Another study also showed that the eukaryotic elongation factor-2 kinase (eEF-2K) was highly expressed in triple-negative breast cancer cells, promoting cell proliferation, migration, and invasion. Results indicated that TQ could inhibit the protein and mRNA expression of eEF-2K. And, TQ could also promote the production of the tumor suppressor miR-603 as well as the inhibition of the NF-*κ*B pathway [73].

Flavonoids were the major active components of *N. glandulifera* seeds to suppress the proliferation of breast cancer in a concentration-dependent way, by inhibiting the phosphorylation of janus kinase 2 protein in the janus kinase/signal transducer and activators of the transcription (JAK/STAT) signal pathway [74]. Compounds **9** and **33** exhibited cytotoxicity on T98G, U837, U251, and GL261 glioma cancer cell lines at 100 *μ*m with cell viability ranging from 29% to 57% [6, 19]. Compound **56** could suppress tumors in mice in a concentration-dependent way after intraperitoneal injection [75]. Compounds **82–84** (IC_50_ range from 15.83 to 17.79 *μ*M) showed a stronger activity against HepG2 cells than **85** (IC_50_ at 36.95 *μ*M) by the MTT assay, owing to their hydroxymethyl moiety. Compound **83** exhibited inhibitory effects against four human tumor cell lines (Bel7402, HepG2, HCT-8, and A549) with IC_50_ values (7.69, 15.83, 11.39, and 20.06 *μ*M) similar to 5-fluorouracil (10.07, 16.42, 6.30, and 14.15 *μ*M), suggesting that the prenyl group could be responsible for mediating the cytotoxicity [35].

### 3.7. Anti-Inflammatory Analgesic Activity

Administration TQ of lipopolysaccharide/interferon*γ-* (LPS/IFN*γ*-) activated BV-2 microglial cells could increase the expression of neuroprotective proteins and decrease the expression inflammatory cytokines and genes of the NF-*κ*B (nuclear factor-kappa B) pathway [76]. TQ suppressed the expression of TNF-*α*, interleukin-1*β* (IL-1*β*), monocyte chemotactic protein 1, and cyclooxygenase (Cox-2) in PDA cells dose- and time-dependently. TQ could also reduce the transport of NF-*κ*B from the endochylema to the nucleus in PDA cells [77]. TQ could significantly decrease the levels of IL-6, IL-1b, TNF-*α*, and prostaglandin E2 (PGE2) but increase the IL-10 levels [78].

In addition, in a study by Hadi et al. on 42 rheumatoid arthritis (RA) patients, *N. sativa* oil supplementation (1 g/day for 8 weeks) led to a significant reduction in serum malondialdehyde (MDA), NO, and IL-10 compared with the placebo group, indicating that *N*. *sativa* oil could improve the inflammation and reducing the oxidative stress in patients with RA [47]. Compounds **8** and **10** inhibited the lipopolysaccharide-induced nitric oxide emergence at 10 *μ*m with inhibition rates 61% and 41%, respectively. Furthermore, compounds **95–98** could inhibit the nitric oxide secretion via lipopolysaccharide-activated RAW 264.7 macrophages [38].

### 3.8. Cardiovascular Activity and Vessel Protection

Compound **30** (at 0.01 *μ*M) could increase the cell viability from 50% to 96% in hypoxia/reoxygenation-induced H9c2 cardiomyocyte. The effect was comparable with verapamil, which was known for its protective effect on cardiomyocytes. [18]. A study showed the protective effects of TQ on the cardiac damage in BALB/c mice. The results demonstrated that TQ played a positive role in the treatment of sepsis-induced cardiac damage [79]. TQ pretreatment could repair the dimethylhydrazine-induced erythrocyte oxidative stress, anaemia, leukocytosis, and thrombocytosis [80]. A study demonstrated that thymoquinone possessed the ameliorative effects upon the pulmonary blood vessels damaged by LPS in a rat model [81].

### 3.9. Gastric Protection

Formation of free radicals and reactive oxygen seem to play a significant role in ulcerative and erosive lesions of the gastrointestinal tract. Therefore, treatments with antioxidants and free radicals scavengers could reduce the I/R-induced gastric mucosal damage. It was found that *N. sativa* seed oil exhibited an elevation in lactate dehydrogenase and lipid peroxide level and reduced the content of SOD and GSH. Results revealed that *N. sativa* seed oil possessed a significant gastroprotective effect, which could be attributed to their FR scavenging ability. [82]. It was also found that TQ had novel gastroprotective mechanisms through suppressing the acid secretion, proton pump, and neutrophil infiltration and increasing the secretion of mucin and nitric oxide [83]. The *N. sativa* aqueous extract could replenish the ethanol-induced decreased gastric mucosal nonprotein sulfhydryl and gastric wall mucus content. It could also increase the gastric acid secretion of pylorus and prevent the formation of necrotic ulcer [84]. In a recent research by Mahvash [48] on 51 *Helicobacter pylori* infected patients with functional dyspepsia, the *N. sativa* treatment group (2 g/day for 8 week) significantly increased the *H. pylori* eradication rate compared with the placebo group. And, *N. sativa* could ameliorate the dyspepsia symptoms such as postprandial fullness, gastric pain, or burning and bloating. In addition, the results of administration of polysaccharide BCPP at 200 mg/kg b.w. for ten days demonstrated 85% healing of gastric ulcers. The increase in PGE2 extracellular signal-regulated kinase-2 (ERK-2) showed that BCPP could induce the PGE-2 synthesis via activating the ERK-2 mediated COX-2 activity. Upregulating the expression in matrix metalloproteinase-2 (MMP-2) and downregulating in MMP-9 indicated an indispensable process of gastric mucosal remodulation [53].

### 3.10. Liver Protection

Compounds **18**, **19**, **21**, **23**, and **24** could ameliorate the triglyceride metabolism in high-glucose-pretreated HepG2 cells [14]. Compound **8** could regulate the glucose consumption mediated by the activation of AMPK, which showed more potent ability than metformin [6]. TQ (10 mg/kg) pretreatment could cause amelioration in the lipid peroxidation level and the activity of SOD and improve the histopathological influence induced by paraquate [85]. The therapeutic effect of *N. sativa* upon the cholestatic liver injury was probably via the attenuation of oxidative stress in the bile duct-ligated rat liver tissues [86]. TQ could reduce the serum bilirubin level in disease conditions leading to amelioration in hyperbilirubinemia and liver toxicity induced by cyclophosphamide [87]. TQ (12.5 mg/kg) pretreatment increased the level of malondialdehyde and nonprotein sulfhydryl (-SH) in the liver induced by CCl_4_, indicating TQ as a protective agent for chemical liver injury. However, high dose of TQ could contribute to oxidative the stress-induced liver injury with LD_50_ at 90.3 mg/mg [88]. A study by A. Khonche et al. [49] on 120 nonalcoholic fatty liver disease (NAFLD) patients showed that the treatment with the mixture (2.5 mL *sativa* seed oil, 1.25 mL honey, and 1.25 mL water/day.) for 3 months could improve liver steatosis and injury and blood levels of TG, LDL, and HDL in the NAFLD patients.

### 3.11. Kidney Protection

Owing to its antioxidant ability, TQ significantly inhibited lipid peroxidation and reduced the activities of SOD, GSHPx, and CAT in the cadmium-treated renal tissue [89]. TQ also played a potential protective role in renal toxicity induced by sodium nitrite, which could be attributed to suppress the oxidative stress and restore the balance between pre- and anti-inflammatory [90]. TQ significantly ameliorated the activities of SOD and GST and parameters levels of TNF-*α*, IL-6, and NADPH oxidase-4 (NOX-4), indicating a potential responsibility for TQ in the dioxyxylene-induced nephrotoxicity [91]. In a study by Mohammad et al. [50] on sixty patients with renal stones, the results demonstrated that *N. sativa* (1 g/day for 10 week) could help to prevent kidney stones and to remove early-forming stones.

### 3.12. Lung Protective Activity

Saponins obtained from *N. glandulifera* significantly prolonged the incubation period of cough, while it increased the phenol red secretion of trachea to show a therapeutic effect on ammonia-induced cough in mice [74]. In comparison with the control group, flavonoids showed a significant relaxant effect, which was weaker than that of theophylline [92].

Furthermore, TQ could attenuate symptoms of asthma mediated by the A2 adenosine receptor [93]. *N. sativa* oil could play a role as a potential centrally respiratory stimulant and mediate via the secretion of histamine with the activation of muscarinic cholinergic and histaminergic mechanisms [94]. In a research by Abdulrahman et al. [51] on 80 asthmatics, it was revealed that *N. sativa* oil supplementation (1 g/day) for 4 weeks could improve the asthma symptoms and pulmonary function.

### 3.13. Nervous System Impact

The exploratory behavior and exercise coordination test of the mice indicated that methanolic and aqueous extracts of *N. sativa* seeds significantly inhibited the central nervous system [95]. *N. sativa* oil exerted a psychostimulative effect and regulated the neurotransmission of dopamine and serotonin, which were a great help of treatment of cognitive disorders [96].

TQ showed the neuroprotective effects on MPP^+^ and rotenone toxicities via rescuing 83–100% of THir neurons in comparison with the rotenone-treated cultures [97]. Moreover, TQ had a neuroprotection potential against A*β*_1–42_ (Alzheimer's amyloid-*β* peptide) toxicity in the rat hippocampal and cortical neurons. Therefore, it might be a potential candidate for Alzheimer's disease treatment [98]. TQ might play an anticonvulsive role in small seizure epilepsy via the opioid receptor-mediated increase of gamma-aminobutyric acid [99].

### 3.14. Diuretics

Administration with the ethanol extract of *N. sativa* (100 mg·kg^−1^) in Wistar Bratislava rats could lead to an increase in the urine volume, which was less than the volume when furosemide was used as a positive drug. The extract of *N. sativa* showed a better natriuretic effect than kaluretic effect. However, for *N. damascena*, the diuretic activity was not due to an increase in the kaluretic effect but mostly to a decrease in Na^+^ excretion [100].

### 3.15. Other Activities

Compounds **76–78** from *N. damascena* showed an estrogenic activity in a dose-dependent manner, suggesting that phenolic compounds contributed to regulate menstruation [5]. Thymoquinone had a positive effect upon postoperative adhesions on an experimental abdominal adhesion model [101].

## 4. Conclusions and Future Prospects

There are more than 20 species of *Nigella* genus, but only *N. glandulifera*, *N. sativa*, and *N. damascena* have been studied. The bioactivities of *N. sativa* seeds are mainly attributed to volatile oils, alkaloids, and steroids, while *N. glandulifera* mostly contains saponins, flavonoid glycosides, and phenolic compounds. Seeds of *Nigella* plants can be used as dietary nutritional supplements, expelling agents, diuretics, and preservatives, playing a positive role in antibacterial, anti-inflammatory, antioxidant, antitumor, and lipid-lowering activities and liver protection on the basis of various biological active substances such as volatile oil, saponin, flavonoids, and alkaloids.

TQ is the major active component in the essential oil of *N. sativa* seeds with anti-inflammatory, antioxidant, antidiabetic, cough and asthma, anticancer, liver protection, and neuroprotective functions. The extract's functions on the basis of single or multiple chemical constituents are ambiguous. Many evidences indicate that TQ should be studied further in the medical treatment. And, further exploration of the pharmacological effect will scientifically explain the traditional application of the seeds of *Nigella*, providing a theoretical basis for the further development and utilization of *Nigella* seeds.

## Figures and Tables

**Table 1:** Chemical constituents of *Nigella* genus.

Compound	Name	Source	Reference
Alkaloids **1–38**			
**1**	Nigeglanine	I	[6, 7]
**2**	4-*O*-Methylnigeglanine	I	[6]
**3**	Nigellicine	I	[7, 8]
**4**	Nigellimine	I	[9]
**5**	Nigellimine *N*-oxide	I	[9]
**6**	Nigellidine	I	[6–8]
**7**	4-*O*-methylnigellidine	I	[6]
**8**	17-*O*-(*β*-D-glc^*p*^)-4-*O*-Methylnigellidine	I	[6]
**9**	Nigelanoid	I	[6]
**10**	Nigeglapine	II	[10]
**11**	Nigeglaquine	II	[10]
**12**	Nigellidine-4-*O*-sulfite	II	[7]
**13**	Nigellaquinomine	II	[11]
**14**	Nigelladine A	II	[11]
**15**	Nigelladine B	II	[11]
**16**	Nigelladine C	II	[11]
**17**	Nigellamine A_1_	I	[12]
**18**	Nigellamine A_2_	I	[12]
**19**	Nigellamine A_3_	I	[13]
**20**	Nigellamine A_4_	I	[13]
**21**	Nigellamine A_5_	I	[13]
**22**	Nigellamine D	I	[14]
**23**	Nigellamines B_1_	I	[12]
**24**	Nigellamine B_2_	I	[12]
**25**	Nigellamine C	I	[13]
**26**	Nigellamine B_3_	I	[14]
**27**	Magnoflorine	I	[15]
**28**	Fuzitine	II	[16]
**29**	Higenamine	II	[17]
**30**	Nigegladine A	II	[18]
**31**	Nigegladine B	II	[18]
**32**	Nigegladine C	II	[18]
**33**	4, 8-Dimethoxy-1-vinyl-*β*-carboline	II	[19]
**34**	7H-Benzo[c]carbazole	II	[19]
**35**	Indolyl-3-carboxylic acid	II	[19]
**36**	Berberine	II	[19]
**37**	Damascenine	III	[20]
**38**	Damascinine	III	[20]
Flavonols **39–55**			
**39**	Quercetin	II	[21]
**40**	Kaempferol	II	[21]
**41**	Rutin	II	[21]
**42**	Kaempferol-3-*O*-*β*-D-glc^*p*^-(1⟶2)-*β*-D-glc^*p*^(1⟶2)-D-glucopyranoside	II	[21]
			[22]
**43**	Nigeglanoside [kaempferol-3-*O*-*β*-D-gal^*p*^-(1⟶3)-*β*-D-glc^*p*^-(1⟶3)-*β*-D-glucopyranoside]	II	[23]
**44**	Kaempferol-3-*O-β*-D-glc^*p*^-(1⟶2)-*β*-D-gal^*p*^-(1⟶2)-glc^*p*^	II	[24]
**45**	Quercetin-3-*O-β-*D-glc^*p*^-(1⟶2)-*β-*D-gal^*p*^-(1⟶2)-glc^*p*^	I	[25]
**46**	Quercetin-3-*O*-(6-*O*)-feroyl-*β-*D-glc^*p*^-(1⟶2)*-β-*D-gal^*p*^-(1⟶2)-glucopyranoside	I	[25]
**47**	Nigelloside	II	[17]
**48**	Kaempferol-3-*O-α-*L-rha^*p*^-(1⟶6)-*β*-D-glc^*p*^(1⟶2)-*β*-D-gal^*p*^-(1⟶2)-*β*-D-glucopyranoside	II	[24]
**49**	Nigelflflavonoside A	II	[26]
**50**	Nigelflflavonoside B	II	[26]
**51**	Nigelflflavonoside C	II	[26]
**52**	Nigelflflavonoside D	II	[26]
**53**	Nigelflflavonoside E	II	[26]
**54**	Nigelflflavonoside F	II	[26]
**55**	Kaempferol-3-*O-β*-D-glucopyranoside	III	[20]
Triterpenoids **56–67**			
**56**	*α*-Hederin	II	[24]
**57**	3-*O-*[*α*-L-rha^*p*^-(1⟶2)-*α*-L-ara^*p*^]-28-*O-*[*β*-D-glc^*p*^]-Hederagenin	II	[24]
**58**	3-*O-*[*α*-L-rha^*p*^-(1⟶2)-*α*-L-ara^*p*^]-28-*O-*[*β*-D-glc^*p*^-(1⟶6)-*β*-D-glc^*p*^]-Hederagenin	II	[24]
**59**	3-*O-*[*α-*L-rha^*p*^(1⟶2)-*α*-L-ara^*p*^]-28-*O-*[*α*-L-rha^*p*^(1⟶4)-*β*-glc^*p*^(1⟶6)-*β*-D-glc^*p*^]-Hederagenin	II	[24]
**60**	3-*O-*[*β*-D-xyl^*p*^-(1⟶3)-*α*-L-rha^*p*^-(1⟶2)-*α*-L-ara^*p*^]-Hederagenin	II	[24]
**61**	3-*O-*[*β*-D-xyl^*p*^-(1⟶3)-*α*-L-rha^*p*^-(1⟶2)-*α*-L-ara^*p*^]-28-*O*-[*β*-D-glc^*p*^]-Hederagenin	II	[24]
**62**	3*-O-*[*β*-D-xyl^*p*^(1⟶3)-*α*-L-rha^*p*^-(1⟶2)-*α*-L-ara^*p*^]-28-*O-*[*α*-L-rha^*p*^-(1⟶4)-*β*-D-glc^*p*^-L-(1⟶6)-*β*-D-glc^*p*^]-Hederagenin	II	[27]
**63**	Hederagenin	II	[28]
**64**	Eleutheroside K	II	[29]
**65**	11-Methoxy-16-hydroxy-17-acetoxy-3-*O-*[*β*-D-xyl^*p*^(1⟶3)-*α*-L-rha^*p*^(1⟶4)-*β*-D-glc^*p*^]-hederagenin	I	[30]
**66**	Lanosterol	I	[31]
**67**	Cyclolaudenol	II	[23]
**Steroids 68–73**			
**68**	*β*-Sitosterol	I II	[23]
**69**	Stigmasterol	I	[32]
		II	[31]
**70**	Camphoral	I	[31]
**71**	Cholesterol	I	[31]
**72**	Ergosterol	I	[31]
**73**	Daucosterol	II	[33]
**Phenolics 74–87**			
**74**	*p*-Hydroxybenzoic acid	I	[25]
**75**	2-Methyl-5-isopropyl-*p*-diphenol	II	[32]
**76**	2,4-Dihydroxyphenylacetic acid	III	[5]
**77**	3,4-Dihydroxy-phenylethanol	III	[5]
**78**	Methyl-2, 4-dihydroxyphenylacetate	III	[5]
**79**	5,7-Dihydroxy-isobenzofuranone	II	[34]
**80**	5,7-Dihydroxy-6-(3-methyl-2-enyl)-isobenzofuranone	II	[28]
**81**	1-O-(2, 4-Dihydroxyphenylacetyl) glycerol	III	[35]
**82**	Nigephenol A	II	[36]
**83**	Nigephenol B	II	[36]
**84**	Nigephenol C	II	[36]
**85**	Salfredin B_11_	II	[36]
**86**	*p*-Coumaric acid	III	[20]
**87**	Caffeic acid	III	[20]
Monoterpenoids **88–94**			
**88**	6-Methoxythymol-3-*O*-*β*-D-glucopyranoside	II	[37]
**89**	[*β*-D-glc^*p*^-(6⟶1)-*β*-D-glc^*p*^]-2-Methyl-5-isopropyl-*p*-pairphenolic glycoside	II	[24]
**90**	*trans*-Sabinene hydrate methyl ether	I	[38]
**91**	*Cis*-Sabinene hydrate methyl ether	I	[38]
**92**	1,2-Epoxy-menth-4(8)-ene	I	[38]
**93**	1,2-Epoxy-menth-4-ene	I	[38]
**94**	Thymoquinone (TQ)	I	[39]
Lipids **95–97**			
**95**	Methyl nonadeca-15, 17-dienoate	I	[40]
**96**	Pentyl hexadec-12-enoate	I	[40]
**97**	Pentyl pentadec-11-enoate	I	[40]
Saccharides **98 & 99**			
**98**	Nigellamose	II	[33]
**99**	Saccharose	II	[33]

I: *N. sativa* seeds; II: *N. glandulifera* seeds; III: *N. damascene* seeds.

**Table 2:** Characteristics of human studies of *Nigella sativa* seeds.

Area	Author (date)	Subjects	Intervention	Dosage	Duration	Results
Amelioration of oxidative stress	Farhangi et al. [45]	Hashimoto's thyroiditis patients (*n* = 23 per group)	*Nigella sativa* capsules	1 g/day	8 weeks	Significant increase in serum TAC, SOD, and reduction in MDA.
Glycemic control	Hebatallah et al. [46]	T2DM patients (*n* = 21 per group)	*Nigella sativa* capsules	1.35 g/day	3 months	Amelioration of IR and significant reduction in alanine aminotransferase (ALT), TC, LDL, HDL, TG, and TAC comparable to metformin.
Anti-inflammation	Hadi et al. [47]	Rheumatoid arthritis patients (*n* = 23 per group)	*Nigella sativa* oil capsules	1 g/day	8 weeks	(1) Significant reduction of serum MDA, NO, and IL-10 compared with that of the placebo group. (2) No significant differences in serum TNF-*α*, SOD, catalase, and TAC compared with that of the placebo group.
Gastric protection	Mahvash et al. [48]	*H. pylori*-infected patients (*n* = 24 per group)	*Nigella sativa* capsules	2 g/day	8 weeks	(1) Significant increase in the *H. pylori* eradication rate compared with that of the placebo group. (2) Significant decrease in dyspepsia symptoms (postprandial fullness, gastric pain, or burning and bloating). (3) Significant increase in dietary-intake, weight, and body mass index and improvement in physical health.
Liver protection	Khonche et al. [49]	Nonalcoholic fatty liver disease patients (*n* = 60 per group)	*Nigella sativa* oil	2.5 mL/day	3 months	(1) Significant improvement in hepatic steatosis compared with that of the placebo group. (2) Significant increase in ALT, AST, LDL, HDL, and TG compared with that of the placebo group.
Kidney protection	Mohammad et al. [50]	Patients with renal stones (*n* = 27 per group)	*Nigella sativa* capsules	1 g/day	10 weeks	Significant reduction in the stone size as compared with the placebo.
Lung protection	Abdulrahman et al. [51]	Asthmatic patients (*n* = 40 per group)	*Nigella sativa* oil capsules	1 g/day	4 weeks	Significant improvement in asthma symptoms and pulmonary functions as compared with the placebo.

## Data Availability

The data supporting this article are from previously reported studies, which have been cited. The data are available from the corresponding author upon request.
